# Favorable Effects of Tacrolimus Monotherapy on Myasthenia Gravis Patients

**DOI:** 10.3389/fneur.2020.594152

**Published:** 2020-10-27

**Authors:** Zhirong Fan, Zunbo Li, Faxiu Shen, Xueping Zhang, Lin Lei, Shengyao Su, Yan Lu, Li Di, Min Wang, Min Xu, Yuwei Da

**Affiliations:** ^1^Department of Neurology, Xuanwu Hospital, Capital Medical University, Beijing, China; ^2^Department of Neurology, Xi'an Gaoxin Hospital, Xi'an, China

**Keywords:** myasthenia gravis, tacrolimus, monotherapy, clinical effectiveness, adverse events

## Abstract

**Background and Purpose:** Tacrolimus (TAC) has been proven to be a rapid-acting, steroid-sparing agent for myasthenia gravis (MG) therapy. However, evidence related to the effectiveness of TAC alone is rare. Therefore, this study was performed to investigate the effect of TAC monotherapy in MG patients.

**Methods:** Forty-four MG patients who received TAC monotherapy were retrospectively analyzed. A mixed effect model was used to analyze improvements in MG-specific activities of daily living scale (MG-ADL), quantitative MG score (QMG) and MG-ADL subscores. Kaplan-Meier analysis was used to estimate the cumulative probability of minimal manifestations (MM) or better. Adverse events (AEs) were recorded for safety analyses.

**Results:** Of the patients receiving TAC monotherapy, MG-ADL scores were remarkably improved at 3, 6 and 12 months compared with scores at baseline (mean difference and 95% CIs: −3.29 [−4.94, −1.64], −3.97 [−5.67, −2.27], and −4.67 [−6.48, −2.85], respectively). QMG scores significantly decreased at 6 and 12 months, with mean differences and 95% CIs of −4.67(−6.88, −2.45) and −5.77 (−7.55, −4.00), respectively. Estimated median period to achieve “MM or better” was 5.0 (95% CIs, 2.8, 7.2) months. Ocular MG (OMG) and generalized MG (GMG) showed similar therapeutic effects in cumulative probabilities of “MM or better” (*P*-value = 0.764). A better response was observed in MG-ADL subscores for ptosis and bulbar symptoms. AEs occurred in 37.5% of patients and were generally mild and reversible.

**Conclusions:** TAC monotherapy is a promising option to rapidly alleviate all symptoms of MG, especially for ptosis and bulbar symptoms.

## Introduction

Immunosuppressive therapies are a major part of standard myasthenia gravis (MG) therapy. It is usually necessary for patients to maintain immunosuppression agent for many years, even for their whole life (1, 2). Corticosteroids are the most common immunosuppressive agents for MG patients. However, long-term therapy of corticosteroids is usually limited by severe adverse events (AEs), mood symptoms and cosmetic problems (3–5). In recent decades, non-steroidal immunosuppressive agents, including azathioprine (AZA), methotrexate (MTX), mycophenolate mofetil (MMF), and cyclosporine A (CsA), have been successfully used in conjunction with corticosteroids to reduce the dose and side effects of corticosteroids (6). However, the relatively slow onset of action of AZA, MTX and MMF, and the severe nephrotoxicity of CsA limits their use in the treatment of MG (7–10).

Tacrolimus (TAC) acts in a manner similar to CsA and exhibits a similar effect to CsA at concentrations 100 times lower (11). Moreover, it has a lower incidence of nephrotoxicity than CsA (12). Several studies have proven that TAC co-administered with corticosteroids can rapidly improve myasthenic symptoms subjectively within 1 month and objectively at 2~3 months (13–15). TAC is recommended to treat MG in different countries and the international MG treatment guidelines (2, 16, 17). Interestingly, four ocular MG (OMG) patients were reported to respond well to TAC alone (18). Meanwhile, CsA monotherapy could significantly improve MG symptoms in RCTs (19, 20). Therefore, we speculate that TAC monotherapy would be a promising option for patients who refuse or cannot tolerate corticosteroids and other immunosuppressive agents.

Herein, we investigated the effectiveness and safety of TAC monotherapy in MG patients. We also analyzed the differential sensitivity to TAC for MG symptoms.

## Materials and Methods

### Participants

Data were collected from the Xuanwu Hospital Capital Medical University Myasthenia Gravis Trial Database from July 01, 2017, to June 01, 2020. A total of 185 MG patients who received TAC therapy were identified. The following exclusion criteria were applied. Patients who had a QMG or MG-ADL score of 0 at baseline were excluded. Any patient who received intravenous immunoglobulin or plasma exchange within 4 weeks prior to the start of TAC administration was excluded. Patients who had undergone thymectomy or received other immunosuppressive agents within 24 weeks prior to the start of TAC administration were excluded. Concurrent use of cholinesterase inhibitors within the usual dosage range was permitted. MG was diagnosed based on a combination of clinical pattern of myasthenia weakness (muscle weakness and fatigability), laboratory tests (positive for anti-AChR or anti-MuSK antibodies), neurophysiological tests (repetitive nerve stimulation) and positive response to acetylcholinesterase therapy.

**Figure 1:**
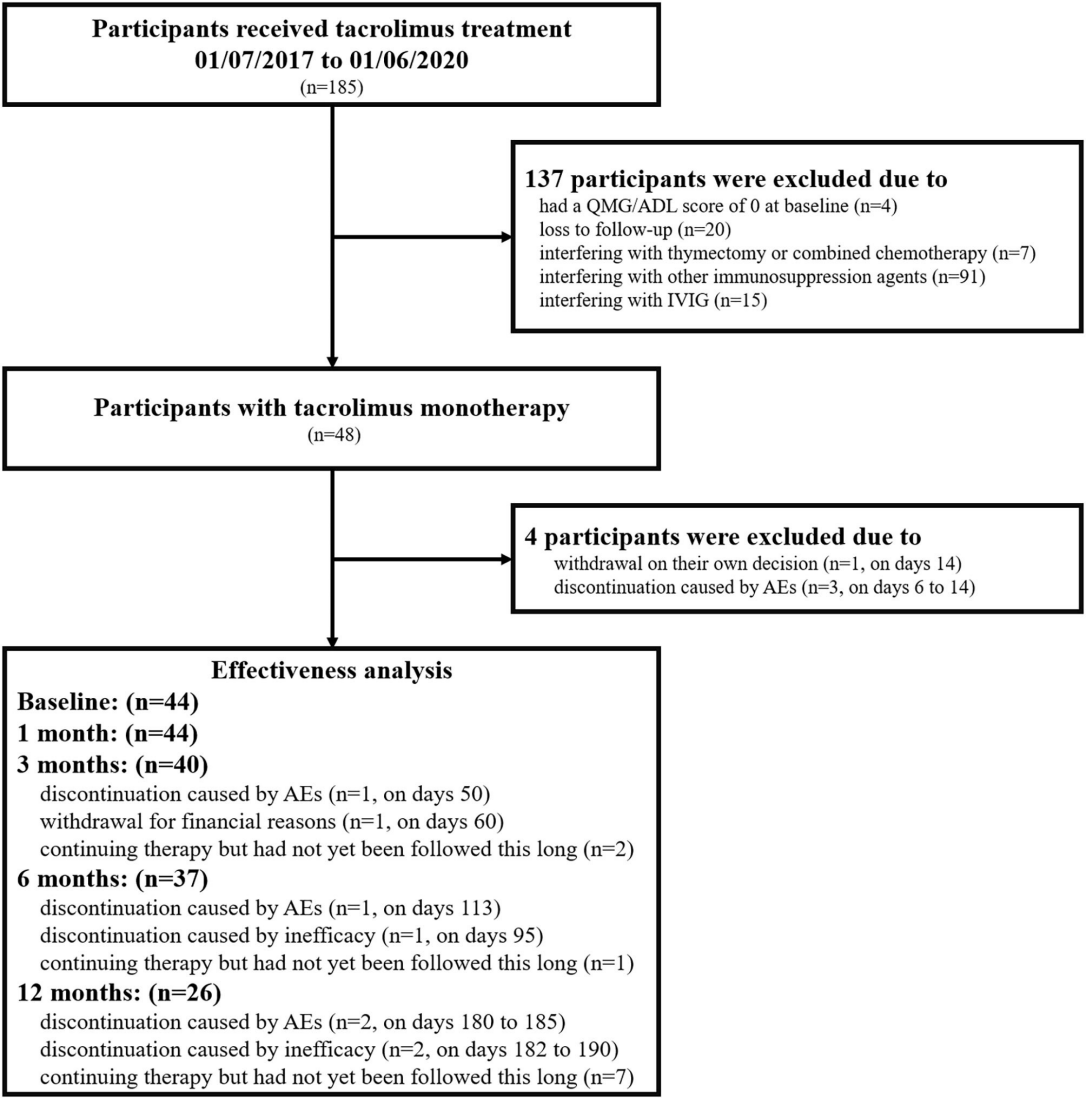
Flowchart of the participants included in the current study. *n*, number of patients; QMG, quantitative myasthenia gravis score; MG-ADL, myasthenia gravis activities of daily living; IVIG, intravenous immunoglobulin; AEs, adverse events.

Finally, we identified 48 patients with TAC monotherapy, for whom corticosteroids or other immunosuppressive agents were contraindicated or refused due to potential AEs. Four patients were excluded from effectiveness analyses who withdrew TAC within 1-month for AEs or patient decisions. Therefore, effectiveness analyses were evaluated in 44 patients, for whom TAC monotherapy was maintained for more than 1 month (Figure 1). Among them, 17 patients with OMG started TAC monotherapy due to inadequate or no response to pyridostigmine (2). The most common problem that limited the use of corticosteroids was contraindications of corticosteroids (28/48), including osteoporosis, poorly controlled hypertension, and diabetes. The remaining 20 patients refused corticosteroids or other immunosuppressive agents due to potential adverse events. The study was approved by the Ethics Committee of Xuanwu Hospital, Capital Medical University, China (No. 2017084) and was in accordance with the principles of the Declaration of Helsinki. Each participant provided written informed consent for participation.

### Tacrolimus Therapeutic Regimens

All patients were treated with an initial daily dose of TAC 2 mg. TAC was increased or Wuzhi tablets were added to achieve adequate TAC concentrations (4.8–10 ng/ml) (21). The maintenance dose ranged from 2 to 4 mg and was adjusted depending on clinical efficacy, side effects, and TAC concentrations. TAC concentrations were commonly measured in whole blood by microparticle enzyme immunoassay.

### Outcome Measurement and Follow-Up

The following characteristics of the patients were collected: sex, age at onset, disease course, serum antibodies, thymus histopathology, Myasthenia Gravis Foundation of America (MGFA) clinical classification, MG-specific activities of daily living scale (MG-ADL), quantitative MG score (QMG), and MGFA post-intervention status (PIS). The MG-ADL scores were assessed by follow-up at 1, 3, 6, and 12 months. A quantitative assessment of muscle strength with QMG scores provided further objective criteria for clinical improvement at 6-month and 12-month face-to-face clinic visits. In terms of PIS, the classification of “MM or better” included minimal manifestations, pharmacological remission, and complete stable remission. Clinical assessment was performed at a fixed interval from the last administration of cholinesterase inhibitor to avoid modification by pyridostigmine.

The therapeutic effects were first evaluated by the improvement in MG-ADL and QMG scores and the probability of achieving “MM or better” during the follow-up period. Next, the probability of achieving “MM or better” was compared between subgroups of OMG and generalized MG (GMG). Then, the differential sensitivity to TAC monotherapy for MG symptoms was investigated by the improvement in MG-ADL subsocres. Safety was assessed by the incidence and severity of AEs and the incidence of AEs leading to drug withdrawal. Renal and liver function injury were assessed by elevation above the upper normal limit of blood urea nitrogen (BUN)/serum creatinine (sCr) and liver enzymes.

### Statistical Analysis

Data of categorical variables were represented as frequencies (%). Data of continuous variables were represented as mean ± standard deviation (SD) or median (interquartile range [IQR]). The linear mixed model for repeated measure analysis was used to compare MG-ADL, QMG scores or MG-ADL subscores among different follow-up periods. The estimates and 95% confidence intervals (CIs) of coefficients in the model were presented to describe changes in MG-ADL, QMG scores or MG-ADL subscores. The model included subjects as a random effect and follow-up period as a fixed effect. Kaplan-Meier analysis was used to estimate the cumulative probability of PIS status “MM or better.” A log-rank test was used for the comparison of treatment outcome between subgroups of OMG and GMG. For patients who withdrew TAC due to ineffectiveness, the last collected data were used as records to be estimated during the remaining periods. No data were included in the effectiveness analyses at a particular time for patients who withdrew TAC due to AEs, failed to have a visit or had not yet been followed this long. Statistical analysis was performed using SPSS version 22.0. The Bonferroni correction was used to decrease the risk of a type I error by adjusting the probability *P*-values. An adjusted value of *P* < 0.05 was considered statistically significant.

## Results

### Patient Characteristics

The demographic characteristics of 44 MG patients (28 males, 16 females) are summarized in Table 1. The mean age at onset was 54.1 ± 17.2 years old, and 29.5% (13/44) of patients had early onset (younger than 50 years old). Thymectomy was performed in eight patients (18.1%). Thirty-eight patients were anti-AChR antibody positive, and one patient was anti-MuSK antibody positive. According to the MGFA classification, there were 17 OMG and 27 GMG patients. The mean dose of TAC was 2.70 ± 0.62 mg, and the mean TAC trough concentration was 6.21 ± 2.59 ng/ml.

**Table 1:** Demographic features of 44 MG patients with tacrolimus monotherapy.

**Demographic characteristics**	
Age at onset (years) (mean ± SD)	54.1 ± 17.2
Early-onset MG^a^/Late-onset MG (*n*)	13/31
Sex(male/female) (*n*)	28/16
**MGFA classification (*****n*****)**	
I	17
II	20
III-IV	7
**Serum antibodies positive (*****n*****)**	
Anti-AchR	38
Anti-MuSK	1
Dual seronegative	5
**Abnormal thymus gland (*****n*****)**	
Thymoma	3
Thymic hyperplasia	4
Thymic cyst	2
Disease course (month) (median [IQR])	10.0 (3.2–20.8)
Tacrolimus dose (mg/day) (Mean ± SD)	2.70 ± 0.62
Tacrolimus trough concentration (ng/ml) (Mean ± SD)	6.21 ± 2.59

a *Onset age was younger than 50 years old*.

*SD, standard deviation; IQR, interquartile range; n, number of patients; MG, myasthenia gravis; MGFA classification, Myasthenia Gravis Foundation of America clinical classification; AChR, acetylcholine receptor; MuSK, muscle-specific tyrosine kinase*.

### Therapeutic Effects of TAC Monotherapy

**Figure 2:**
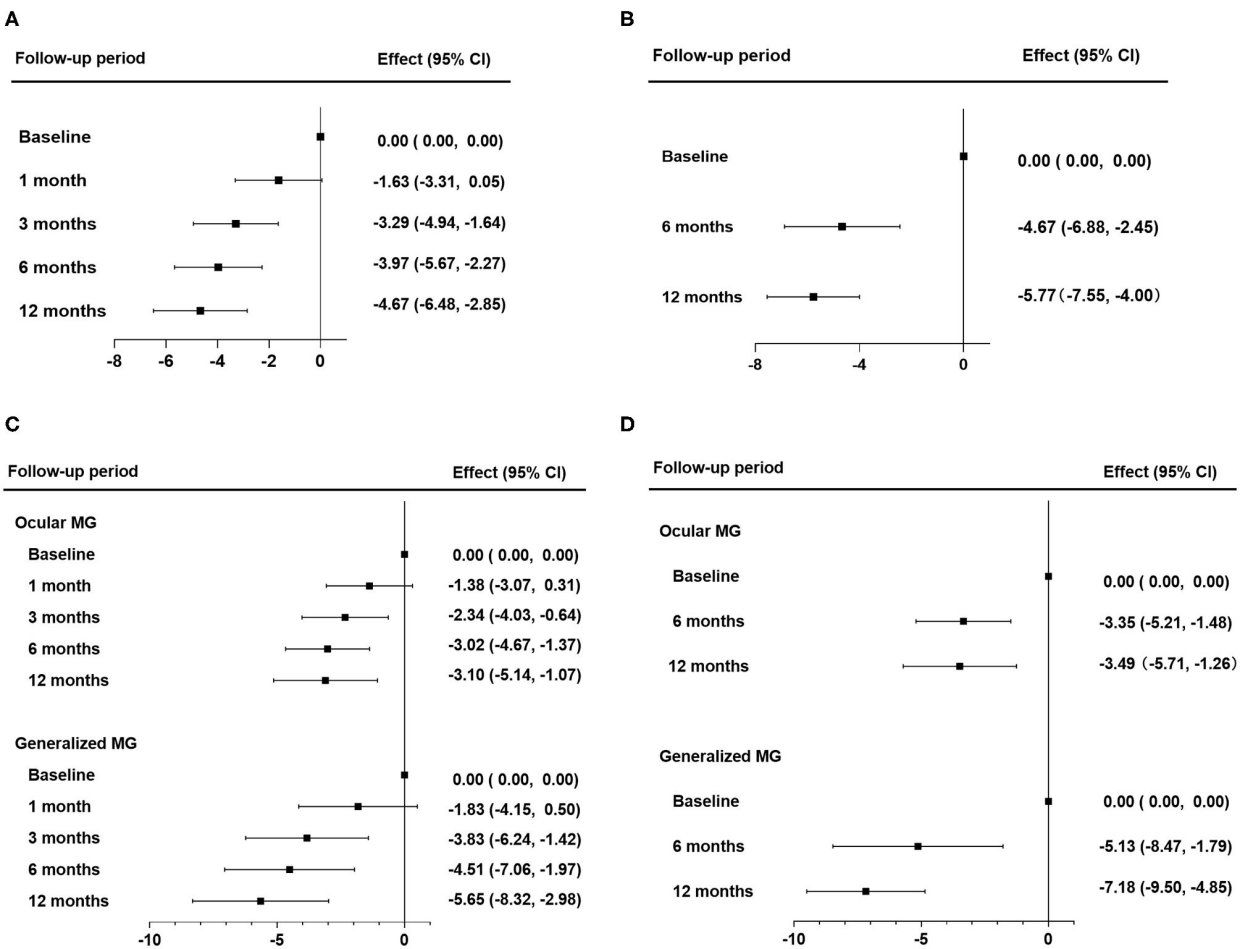
Therapeutic effects of tacrolimus monotherapy. **(A,B)** Therapeutic effects in all patients were evaluated by changes in MG-ADL scores **(A)** and by changes in QMG scores **(B)**. **(C,D)** Therapeutic effects in subgroups of OMG and GMG were evaluated by changes in MG-ADL **(C)** and by changes in QMG scores **(D)**. The effect was the mean difference of MG-ADL or QMG scores during the follow-up periods. Statistical analysis was performed by linear mixed model for repeated measurements with Bonferroni correction. QMG, quantitative myasthenia gravis score; MG-ADL, myasthenia gravis activities of daily living; OMG, ocular myasthenia gravis; GMG, generalized myasthenia gravis.

The median values of MG-ADL and QMG scores during follow-up periods are shown in Table 2. The linear mixed model for repeated measurements showed significant improvements in both MG-ADL and QMG scores. Scores of MG-ADL at 3, 6, and 12 months were significantly lower than those at baseline, with mean differences and 95% CIs of −3.29 ([−4.94]–[−1.64]), −3.97 ([−5.67]–[−2.27]), and −4.67 ([−6.48]–[−2.85]), respectively (Figure 2A). Scores of QMG at 6 and 12 months significantly decreased compared with scores at baseline, in which the mean difference was −4.67 (95% CIs, [−6.88]–[−2.45]) and −5.77 (95% CIs, [−7.55]–[−4.00]), respectively (Figure 2B). Remarkable improvements in MG-ADL and QMG scores were observed in both OMG and GMG patients, and were beginning at 3 months in MG-ADL scores (Figures 2C,D).

**Table 2:** Scores of MG-ADL and QMG during follow-up periods.

	**Scores, median (IQR)**				
	**Baseline**	**1 month**	**3 months**	**6 months**	**12 months**
**MG-ADL**					
Overall	6 (4–7.75)	4 (2.75–6)	3 (1–4)	2 (0–3.25)	1 (0–2)
In OMG	5 (3–6)	3(2–5)	2.5 (1–3.75)	1.5 (0–3)	1 (0–3)
In GMG	7 (5–10)	5(4–7)	3 (1–4)	2 (0–4)	0.5 (0–2)
**QMG**					
Overall	7 (6–10)	-	-	3 (1–6)	3(1–3)
In OMG	6 (4–6)	-	-	1 (0.5–3.5)	1(0–3.75)
In GMG	10 (7–11.5)	-	-	5.5 (1–6.25)	3(1–3)

*IQR, interquartile range; MG-ADL, myasthenia gravis-specific activities of daily living scale; QMG, quantitative myasthenia gravis score; MG-ADL, myasthenia gravis activities of daily living; OMG, ocular myasthenia gravis; GMG, generalized myasthenia gravis*.

**Figure 3:**
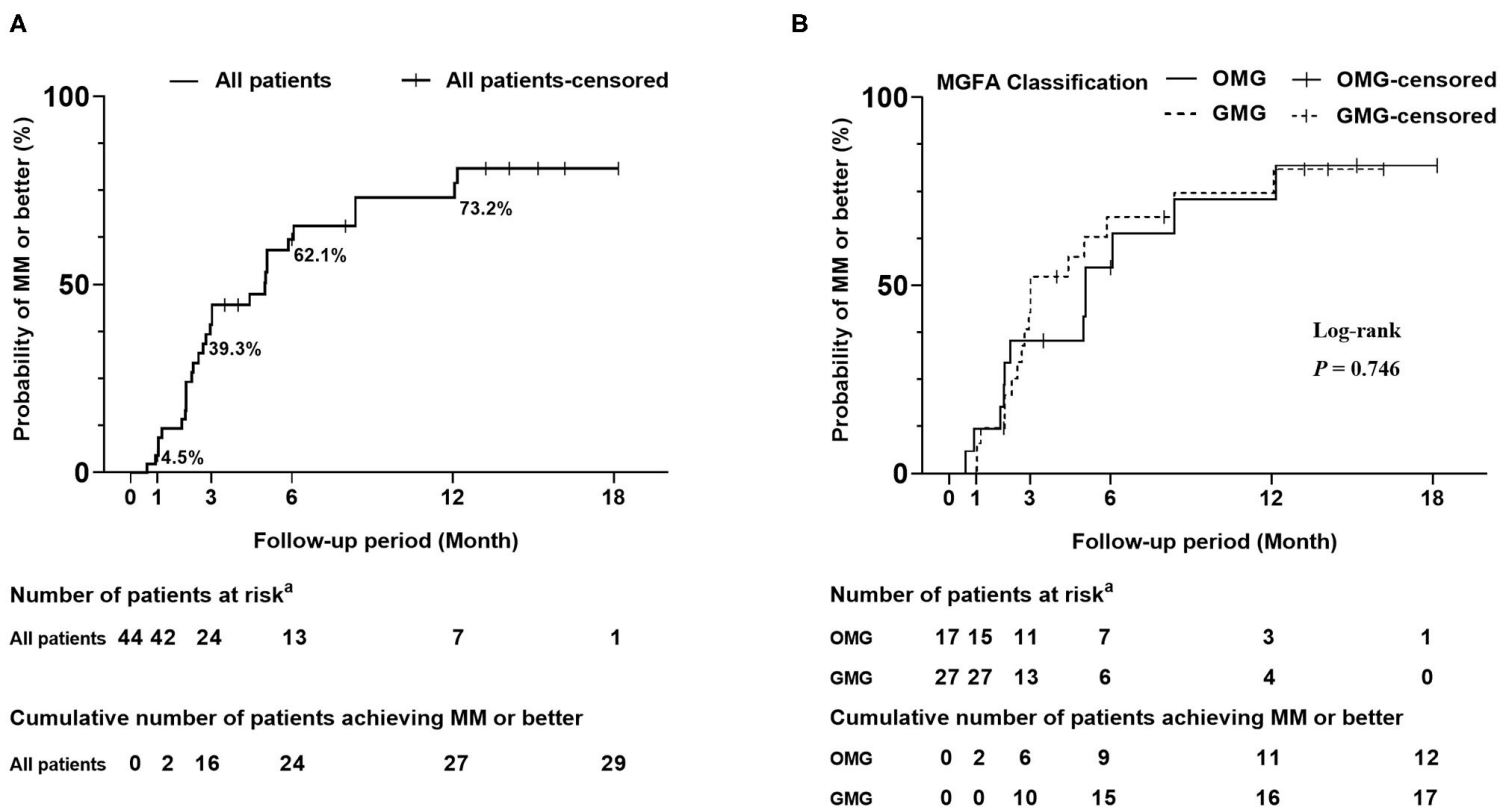
Kaplan-Meier curves for the cumulative probability of achieving “MM or better”. **(A)** Cumulative probability of achieving “MM or better” in all patients. The cumulative probability of achieving “MM or better” was 4.5, 39.3, and 62.1% at 1 month, 3 months, and 6 months, respectively. Finally, it rose to 73.2% at 12 months. **(B)** Cumulative probability of achieving “MM or better” in subgroups of OMG and GMG. A log-rank test was used for the comparison of treatment outcome between subgroups of OMG and GMG. Crossing marks indicated censoring time; “MM or better” included MGFA post-intervention status of minimal manifestations, pharmacological remission and complete stable remission. ^a^Number of patients who were still in the study and did not achieve “MM or better” at the end of specified time. OMG, ocular myasthenia gravis; GMG, generalized myasthenia gravis.

Up to 84.1% of patients reported subjective improvement within the first month. The cumulative probability of achieving “MM or better” in all patients showed a gradual increase and rose to 73.2% at 12 months (Figure 3A). The estimated median period to achieve “MM or better” was 5.0 (95% CIs, 2.8–7.2) months. Kaplan–Meier analysis showed no differences in cumulative probabilities between OMG and GMG (*P*-value = 0.764) (Figure 3B). The estimated median periods were 5.1 (95% CIs, 3.9–6.2) and 3.0 (95% CIs, 1.0–5.1) months for OMG and GMG, respectively.

The treatment was discontinued in 1 patient on days 95 and 2 patients on days 182 to 190 because it was judged to be ineffective by the treating physician (Figure 1). No patients experienced exacerbation or developed a crisis during the follow-up period.

### Differential Sensitivity of TAC Monotherapy for MG Symptoms

**Figure 4:**
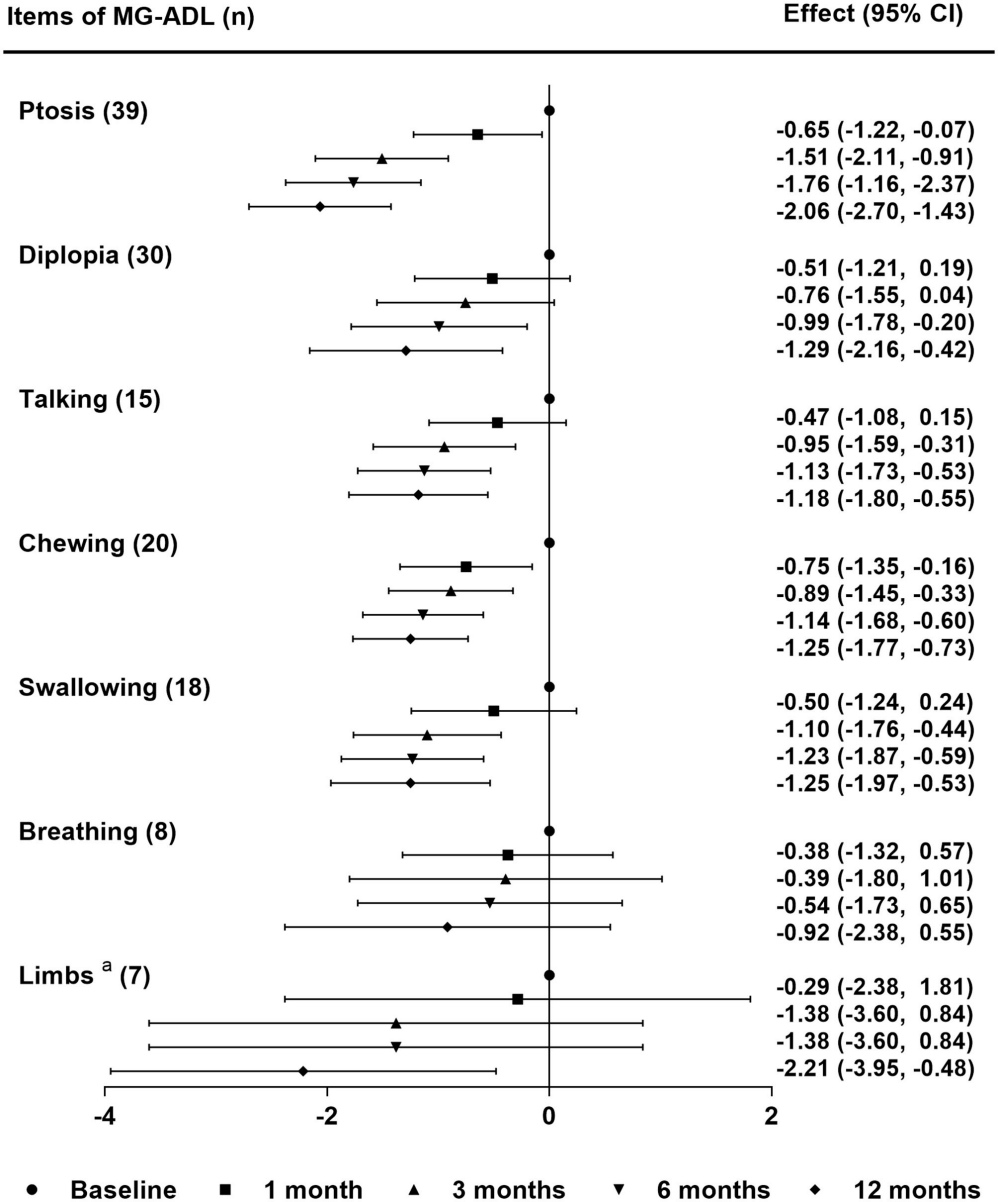
Differential sensitivity of tacrolimus monotherapy for MG symptoms. The effect was the mean difference of MG-ADL subscores in each symptom during the follow-up periods. Statistical analysis was performed by linear mixed model for repeated measurements with Bonferroni correction. ^a^Limbs included upper and lower limbs. MG-ADL, myasthenia gravis-specific activities of daily living scale; n, number of patients with the symptom at baseline.

A total of 68.2% of patients (30/44) reported subjective improvement of ptosis within the first month. The median values of MG-ADL subscores during follow-up periods are shown in Table 3. The linear mixed model showed significant improvements in MG-ADL subcores for ptosis and chewing from the first month and for talking and swallowing from 3 months (compared with baseline, *P*-value < 0.05) (Figure 4). For the symptoms of diplopia and limbs, subscores of MG-ADL showed no significant improvement until 6 months and 12 months, respectively. For breathing difficulty, no significant improvement was observed during the follow-up periods (Figure 4). More importantly, all symptoms in all patients were improved or stable, and there was no exacerbation within 12 months. An interesting finding in this study was that 25 patients (56.8%) complained of photophobia or light sensitivity along with the onset of MG symptoms. Among them, 44% of patients (11/25) achieved clinical improvement and 16% of patients (4/25) got remission in photophobia after 6 months treatment. Almost all of patients with photophobia had symptom of ptosis (24/25). Five of these patients (20.8%) reported clinical improvement of photophobia earlier than or along with the improvement of ptosis.

**Table 3:** MG-ADL subscores for MG symptoms during the follow-up periods.

**Items of MG-ADL (*n*)**	**Subscores, median (IQR)**				
	**Baseline**	**1 month**	**3 months**	**6 months**	**12 months**
Ptosis (39)	3 (2–3)	2 (1–3)	1 (0–2)	0 (0–2)	0 (0–1)
Diplopia (30)	2 (1–3)	1 (1–2.5)	1 (0–2.5)	1 (0–2)	0 (0–1.5)
Talking (15)	1 (1–2)	1 (1–1)	0 (0–1)	0 (0–1)	0 (0–0.5)
Chewing (20)	1 (1–2)	1 (0–1)	1 (0–1)	0 (0–1)	0 (0–0)
Swallowing (18)	1 (1–2)	1 (0–1.25)	0 (0–1)	0 (0–0)	0 (0–0)
Breathing (8)	1 (1–1.75)	1 (0.25–1)	1 (0–2)	1 (0–1)	0 (0–0.5)
Limbs^a^ (7)	2 (2–4)	2 (1–4)	1.5 (0–2.25)	1.5 (0–2.25)	0 (0–1.25)

a *Limbs included upper and lower limbs*.

*MG-ADL, myasthenia gravis-specific activities of daily living scale; n, number of patients with the symptom at baseline*.

### Safety of TAC Monotherapy

The incidence of AEs was 37.5% (18/48), and all observed AEs are shown in Supplementary Table 1. The most frequent AEs were BUN/sCr elevation (4/48, 8.33%) and liver enzyme elevation (3/48, 6.25%). Two patients experienced joint pain, which has never been reported in MG patients with TAC therapy before. Four patients who had BUN/sCr elevation were relatively old (the median age was 68.5 years old). All of them had a long history of hypertension (range 5–22 years), and two of them had diabetes mellitus. AEs that led to therapy discontinuation occurred in seven patients on days 6 to 185 (Figure 1). All AEs were mild and resolved after dose reduction or drug withdrawal. No deaths occurred during the follow-up period.

## Discussion

Our study showed that TAC monotherapy could significantly reduce MG-ADL and QMG scores, and induce remission of various symptoms in both OMG and GMG patients. A better prognosis and a more rapid onset of action were observed for ptosis and bulbar symptoms (talking, chewing, and swallowing) than for diplopia, dyspnea, and limb weakness. In addition, side effects were mild and reversible during the 12-month follow-up period. These results demonstrated that TAC monotherapy was an effective and safe therapeutic option for MG patients who refuse or have contraindications to corticosteroids and other immunosuppressive agents.

TAC monotherapy had favorable effects on the outcome of most patients in our study. TAC has been proven to be a steroid-sparing immunosuppressive agent (22) and showed significant improvement in MG-ADL and QMG scores in combination with corticosteroids (13, 23). In our study, among 44 patients who took TAC alone, 84.1% reported improvement within the first month. The MG-ADL scores improved significantly at 3 months in both the OMG and GMG. Six months later, more than 65% of individuals achieved “MM or better.” Monotherapy with TAC in MG patients showed similar rapid onset of action, clinical effectiveness and stable remission to those in whom TAC was co-administered with corticosteroids (13–15). Our results suggested that TAC monotherapy is a reasonable option for patients who refuse or have contraindications for corticosteroids or other immunosuppressive agents.

The sensitivity to TAC monotherapy for various symptoms was differential. Wakata reported that the symptoms of lower extremities, grip strength, ptosis and swallowing responded well to TAC in combination with corticosteroids (24). The percentage improvement in the non-facial composite (arm and leg outstretch times, grip, forced vital capacity, and head lift) was less than that for vision and facial (ptosis, diplopia, swallowing, and chewing) with CsA alone or CsA co-administered with corticosteroids (19, 20). In our study, a better response and a more rapid onset of action were observed for ptosis and bulbar symptoms (talking, chewing, swallowing) than for diplopia, dyspnea, and limb weakness. Therefore, TAC monotherapy could be recommended as the initial treatment for GMG patients with bulbar symptoms and for OMG patients with ptosis who had an inadequate response to pyridostigmine. The clinical response for diplopia was insufficient until 6 months. Accordingly, for young patients with diplopia who refuse corticosteroids due to potential AEs or cosmetic problems, doctors are suggested to persuade them to accept corticosteroids or in conjunction with TAC to achieve adequate responses quickly. Several studies demonstrated that early stages of disease, thymoma and adequate TAC concentration were associated with responsiveness to TAC co-administered with corticosteroids (21, 25). Differential sensitivity for MG symptoms might be a potential predictive factor for effectiveness of TAC monotherapy. Therefore, multiple regression analysis with a larger sample size is required for further precision medicine.

The most interesting finding in our study was that 56.8% (25/44) of patients complained of photophobia or light sensitivity. They had to wear sunglasses, indicating that the intraocular muscles were involved in these patients. Slow pupillary responses and fatigability to light in MG have been reported in several studies (26, 27). The symptoms of photophobia or light sensitivity in our patients could be caused by dysfunction of the pupillary light reflex in bright sunlight. The pupil cycle time technique of Miller and Thompson (26), as a measurement of pupillary responses to light, would be needed to demonstrate the correlation between pupillary dysfunction and the symptom of light sensitivity in further studies. Two smooth muscles, the sphincter muscle, and the dilator muscle, comprise the iris and determine pupil dynamics (28). Lu reported pupillary dysfunction in MG not only involved the sphincter muscle but also the dilator muscle (27). Muscarinic and nicotinic AChR receptors are localized in the sphincter muscle and the dilator muscle, respectively (29). Most cases of MG were positive in antibodies against nicotinic AChR receptors. Antibodies against muscarinic AChR receptors have been detected in MG patients (30). Damaged muscarinic AChR receptors in sphincter muscle and nicotinic AChR receptors in dilator muscle might be the pathogenic mechanism of photophobia. However, the pathogenicity remains to be established. After MG treatments, symptoms of photophobia improved in 60% of patients, which indicated that photophobia in these patients was related to MG. Other common conditions associated photophobia, including ophthalmological pathology, neurological disorders, psychiatric disorders or drugs, should be considered in the remaining 40% of patients who had a poor prognosis of photophobia (31). Most of them only had mild weakness of the orbicular eye muscle instead of difficulties in closing eyes. Typical symptoms of conjunctival infections were absence during the follow-up visits. Unfortunately, none of these patients had records of periodic ophthalmological evaluation. It is unclear whether they had visual refraction defects or cataract in our retrospective study. Therefore, periodic ophthalmological evaluations should be concerned in patients with poor prognosis of photophobia. The improvement of ptosis may concern with photophobia in part of patients. However, the alleviation of photophobia in most patients was delayed than that of ptosis. Thus, ptosis and photophobia should be evaluated as two separated symptoms.

Monotherapy with TAC in 48 MG patients showed a favorable safety profile. Surprisingly, a different profile of AEs was found, contrasting with previous studies, in which TAC was usually co-administered with corticosteroids (22, 23, 32, 33). There were no reports of AEs linked to nephrotoxicity in most previous MG studies with TAC (22, 23, 33). BUN or sCr elevation was found in 8.33% of patients in our study, partly because physicians paid close attention to renal damage of TAC and reported AEs once the elevation was above the upper normal limits. It has been reported that TAC nephrotoxicity originates from a strong vasoconstrictive effect. A high TAC whole-blood concentration and a history of hypertension could increase the risk of renal damage in organ transplantation patients (34). All four patients with mildly elevated BUN/sCr in our study were of old age and had a long history of hypertension, although TAC whole-blood concentrations were much lower than those in organ transplantation. Thus, old MG patients with long-term hypertension need more frequent monitoring of subclinical renal damage and renal function when using TAC. Another AE, reversible joint pain, was found for the first time in two patients in our study. Joint pain is a rare but debilitating AE in organ transplantation patients with TAC treatment. It gradually receded after TAC withdrawal. This phenomenon was named calcineurin inhibitor-induced pain syndrome (CIPS) (35). The reason was suspected as a calcineurin inhibitor-induced vascular disturbance of bone perfusion and permeability causing bone marrow edema (35). The final diagnosis of CIPS requires further examinations, including bone mineral density tests, bone scintigraphy and magnetic resonance imaging (36).

There are several limitations in this study. First, the retrospective nature, a relatively small sample size and no controlled group for comparison weakened the evidences of our results. First, the limited sample size of patients who had dyspnea or limb weakness provided inadequate evidence of sensitivity for these symptoms. Second, loss to follow-up was reported in eight patients in effectiveness analysis. Among them, the therapeutic effects were estimated according to the last records in four patients who withdrew TAC due to ineffectiveness. It was plausible that attrition bias associated with loss to follow-up drove either overestimation or underestimation of therapeutic effect. Last, most patients evaluated had either OMG or mild GMG (MGFA clinical classification type II). There were only seven patients classified as MGFA type III or IV. More patients with MGFA type III or IV or high-quality RCT trials will be needed to further prove the efficacy of TAC monotherapy in MG patients.

## Conclusions

TAC monotherapy is a fast-acting and efficacious regimen to alleviate all common symptoms of both OMG and GMG, especially for patients with ptosis and bulbar symptoms. Close monitoring of renal function is essential for older patients with hypertension.

## Data Availability Statement

The data that support the findings of this study are available from the corresponding author upon reasonable request.

## Ethics Statement

The studies involving human participants were reviewed and approved by Ethics Committee of Xuanwu Hospital, Capital Medical University, China (No. 2017084). Written informed consent to participate in this study was provided by the participants' legal guardian/next of kin.

## Author Contributions

ZF contributed with drafting and revising the manuscript, study concept and design, acquisition of data, and statistical analysis. ZL contributed with drafting and revising the manuscript, study concept and design. FS, XZ, LL, and SS contributed with acquisition of data. YL, LD, MW, and MX contributed with revising the manuscript and interpretation of the data. YD contributed with drafting and revising the manuscript, study concept and design, and interpretation of the data. All authors contributed to the article and approved the submitted version.

## Conflict of Interest

The authors declare that the research was conducted in the absence of any commercial or financial relationships that could be construed as a potential conflict of interest.

## Abbreviations

MG: myasthenia gravis
OMG: ocular myasthenia gravis
GMG: generalized myasthenia gravis
CsA: cyclosporine A
TAC: tacrolimus
AEs: adverse events
MGFA: Myasthenia Gravis Foundation of America
MG-ADL: MG-specific activities of daily living scale
QMG: quantitative MG score
MGFA-PIS: MGFA post-intervention status
IQR: interquartile range
95%CI: 95 % confidence intervals
CIPS: calcineurin-inhibitor induced pain syndrome.
