# Supplementary material for: Favorable Effects of Tacrolimus Monotherapy on Myasthenia Gravis Patients
Source: Front Neurol. 2020 Oct 27;11:594152. doi: 10.3389/fneur.2020.594152 (PMC7652845; doi:10.3389/fneur.2020.594152)
Supplement: Supplementary file 1 [file Table_1.DOCX]

Supplementary Material

**Table S1. Summary of potential drug-related events reported in this study**

| Adverse events | n (%) |
| --- | --- |
| Patients with any adverse events | 18/48(37.5%) |
| Discontinued therapy due to adverse events | 7/48(14.6%) |
| Total number of adverse events observed | 21 |
| BUN/sCr elevation | 4 ^a^ |
| Liver enzyme elevation | 3 ^b^ |
| Tremor | 2 |
| Nausea | 2 |
| Diarrhea | 2 ^b^ |
| Joint pain | 2 ^b^ |
| Abdominal pain | 2 ^b^ |
| Dizziness | 1 |
| Skin itch | 1 |
| High blood pressure | 1 |
| Blood glucose increased | 1 |
| Blood uric acid increased | 1 |
| Discomfort of cardiac origin | 1 ^b^ |

^a^ Two patients discontinued therapy due to this adverse event;

^b^ One patient discontinued therapy due to this adverse event.

BUN = blood urea nitrogen; sCr = serum creatinine; n = number of patients.
